# Fibrotic Scar in Neurodegenerative Diseases

**DOI:** 10.3389/fimmu.2020.01394

**Published:** 2020-08-14

**Authors:** Nadia D'Ambrosi, Savina Apolloni

**Affiliations:** Department of Biology, Tor Vergata University, Rome, Italy

**Keywords:** Alzheimer's disease, amyotrophic lateral sclerosis, astrocytes, fibroblasts, microglia, multiple sclerosis

## Abstract

The process of uncontrolled internal scarring, called fibrosis, is now emerging as a pathological feature shared by both peripheral and central nervous system diseases. In the CNS, damaged neurons are not replaced by tissue regeneration, and scar-forming cells such as endothelial cells, inflammatory immune cells, stromal fibroblasts, and astrocytes can persist chronically in brain and spinal cord lesions. Although this process was extensively described in acute CNS damages, novel evidence indicates the involvement of a fibrotic reaction in chronic CNS injuries as those occurring during neurodegenerative diseases, where inflammation and fibrosis fuel degeneration. In this mini review, we discuss recent advances around the role of fibrotic scar formation and function in different neurodegenerative conditions, particularly focusing on the rising role of scarring in the pathogenesis of amyotrophic lateral sclerosis, multiple sclerosis, and Alzheimer's disease and highlighting the therapeutic relevance of targeting fibrotic scarring to slow and reverse neurodegeneration.

## Introduction

Fibrosis identifies a condition marked by an increase of interstitial fibrous tissue in the parenchyma, induced by an uncontrolled inflammatory reaction derived by a wound healing response to tissue injury. While wound healing represents a necessary action to contain and repair damage, the cellular and molecular events characterizing the fibrotic response can evolve in time and can lead to the distortion of tissue architecture, followed by a loss of organ function with pathophysiological consequences that may even be severe (1, 2). The wound healing response, resulting from neurodegeneration, recruits local and infiltrating immune cells, as well as extracellular matrix (ECM)-producing stromal fibroblasts and astrocytes. Typically, damaged neurons are not replaced by tissue regeneration, and scar-forming cells can persist chronically in brain and spinal cord lesions. Fibrotic scarring, also called mesenchymal scarring, represents the central core of the CNS acute lesions and it mainly consists of endothelial cells and inflammatory immune cells, including monocyte-derived macrophages, stromal fibroblasts, and ECM deposits. The fibrotic core is closely bordered by the so-called glial scar, mainly consisting of astrocytes. CNS responses to acute lesions can be divided into partially overlapping but functionally distinct temporal phases: cell death and inflammation, cell proliferation and tissue replacement, ECM degradation, an tissue remodeling. Chronic injuries of the CNS, occurring in most neurodegenerative diseases, do not display and overt fibrotic condition. Major differences with acute injuries concern the lower intensity of the initial damage which accumulates only gradually, and, as it becomes more severe gives rise to small individual lesions displaying reactive gliosis, multicellular responses and ECM deposits, similarly to acute damages, albeit in a smaller scale, in a wider time-range and in an interspersed manner (3, 4). Pieces of evidence indeed indicate the involvement of a fibrotic reaction in chronic diseases, such as activation of cells of mesenchymal origin, astrocytes, and a redefinition of the ECM. Specifically, myofibroblasts (mainly deriving from endothelial vasculature, meninges, pericytes or infiltrated stromal cells), astrocytes and macrophages contribute to the disproportionate deposition of connective tissue matrix proteins, consisting predominantly of fibronectin, collagen and laminin, as well as glycosaminoglycans (GAGs) (5, 6) that delay tissue repair by stimulating further scarring and fibrosis through communication with inflammatory cells (7, 8). ECM components form dense lattice-like structures surrounding neurons, termed perineuronal nets (PNNs), that in the long-term hamper neural plasticity and axon regeneration and growth (9).

Rising evidence supports a double, and apparently contrasting, role of the CNS scar, in both promoting tissue protection as well as in inhibiting repair. Indeed, scar-forming astrocytes have been extensively studied and regarded as one of the main sources of the axon growth inhibitory mechanism (10) by acting as a physical barrier that delays rather than supporting axon regeneration. At the injury margins, reactive astrocytes reorganize their structure, becoming hypertrophic, with elongated overlapping processes, and display a strong upregulation of intermediate filament proteins such as glial fibrillary acidic protein (GFAP), vimentin and nestin (1). In addition, in the site of CNS damage, reactive macrophages and microglia play crucial roles in driving secondary injury through a vicious neuroinflammatory cycle. Indeed, ECM molecules released by reactive cells activate receptors on macrophages and microglia to induce a pro-inflammatory phenotype that leads to further astrocytic reactivity and matrix molecules deposition. It is reported that in neurodegenerative diseases including amyotrophic lateral sclerosis (ALS), multiple sclerosis (MS), and Alzheimer's disease (AD), activated microglia, secreting pro-inflammatory cytokines, induce the so called “A1” reactive astrocytes. These astrocytes fail to support the survival and differentiation of neuronal cells, and start to drive neuron and oligodendrocyte death (11). Conversely, it has also been reported that astrocytic scar formation may not only have a detrimental role, but it also helps CNS axonal regeneration by forming permissive bridges *in vivo*, known as glial bridges, along which injured CNS axons can regrow and cross the scar when stimulated with appropriate growth factors and by transcriptional activation of neuronal-intrinsic growth pathways (12). For instance, reactive astrocytes appearing after a stroke acquire a repairing phenotype, characterized by the upregulation of neurotrophic factors expression and by the translocation of mitochondrial particles to damaged neurons (13).

Microglia and macrophages promote tissue remodeling and repair by clearance of cellular and myelin debris, degradation of scar tissue and production of neurotrophic factors (14). Depending on the time/phase of the disease, and type of injury (acute vs. chronic) these cells can be involved in a composite response. They participate in secondary tissue damage with consequent glial scar formation, and at the same time they assume an anti-inflammatory phenotype with increased phagocytic activity, producing growth factors and anti-inflammatory cytokines and stimulating tissue repair and regeneration (15). Therefore, a categorization of glial functions is an oversimplification, since their responses (16), characterized simultaneously by both detrimental and protective features, are closely linked and mutually dependent.

Besides astrocytes and microglia, reactive NG2-glia are reported to contribute to the formation of the scar, migrating toward the site of injury and increasing the proliferation and the expression of ECM molecules, as proteoglycans. Moreover, oligodendrocytes precursor cells change their gene expression following CNS damage, starting to express cytokines, and perpetuating the immune response. On the other hand, oligodendrocyte progenitor cells participate in the resolution of the scar, limiting the extent of neurotoxic inflammatory lesion core cells (17).

Recent evidence has shown that, following CNS injury, pericytes, perivascular cells located on microvessels, partake in the fibrotic scar formation by proliferating and differentiating into scar-forming myofibroblasts (18–20).

This mini review aims at summarizing and discussing the current evidence regarding the role of fibrotic scar in the context of neurodegenerative diseases.

## Fibro-Glial Scar in ALS

Amyotrophic lateral sclerosis (ALS) is characterized by motor neuron degeneration in the motor cortex, brainstem, and anterior horns of the spinal cord. Motor neuron loss is a complex phenomenon, where different cell types actively contribute to the pathological mechanism, establishing a non-cell autonomous disease, implying a comprehensive analysis to understand how changes in the function of individual cell types can affect the behavior of other cells. In the CNS, motor neuron loss is indeed accompanied by glial cells activation, oligodendrocyte pathology and toxicity, blood-brain and -spinal barrier permeabilization, and peripheral immune cells infiltration (21). Astrocytosis has a particular impact on the disease, since it is characterized by a massive response of hypertrophic protoplasmic astrocytes surrounding degenerating motor neurons, and intense fibrotic astrocyte reactivity in the white matter (22). This process involves numerous molecular changes toward a reactive phenotype, such as production and secretion of pro-inflammatory cytokines, chemokines, and growth factors, in particular, IL-6, CXCL1, 10, 12, tumor necrosis factor-α, transforming growth factor β (TGFβ), nerve growth factor, interferon γ, prostaglandin D2 (23), as well as ECM components (24, 25). During ALS, such activation could have the protective purpose to circumvent the degeneration spreading and to restrict inflammation by contrasting the infiltration of active immune cells into the injured tissue, preventing further tissue damage. However, the presence of glial scarring, excessive microgliosis and accumulation of ECM into a disorganized PNN structure around motor neurons, actually form a non-permissive environment that is hostile to neuron survival and regeneration, thus resulting in a harmful reaction (26). Therefore, the fibro-gliotic response could have a dual role, where the contribution of the two processes is continuously remodeled over time, eventually shifting the subtle equilibrium between two possible opposite outcomes toward a detrimental one.

It has been demonstrated that in tissues derived from ALS patients and from the SOD1-G93A mouse model, increased levels of TGFβ correlate with disease progression. Persistent elevated amounts of TGFβ are supposed to be responsible for a decrease in neurogenesis, pro-inflammatory reactions, and fibrosis. This latter might promote the progression of ALS indirectly by replacing areas of motor neuron loss with excessive scar tissue. In this respect, mainly astrocytes and microglia produce and release TGFβ that may act on myofibroblasts precursors to induce a profibrotic phenotype (27, 28). Indeed, the motor cortex of ALS patients displays significantly increased levels of fibronectin and collagen IV, indicating fibrotic activity (27). Moreover, the ECM characterizing spinal cord in ALS H46R rats is also increased in chondroitin sulfate proteoglycans (CSPGs), which are supposed to behave as hindering matrices toward any cell-restorative therapy with both cell transplantation and endogenous neural progenitor activation (26). The analysis performed with confocal Raman spectra of spinal cord tissues from SOD1-G93A mice shows that the signature of the gray matter, both in early symptomatic and presymptomatic mice, is markedly different from the one obtained from healthy mice. In addition to axon demyelination and loss of lipid structural order, these spectra differences account for proliferation and aggregation of branched CSPGs that, moreover, appear to be early events in the progression of the disease (29). Furthermore, CSPGs receptors are increased in reactive astrocytes from diseased rats, and this may contribute to further inhibition of neuronal regeneration, through a signaling mechanism induced by CSPGs (25). Accumulation of ECM is moreover testified by the downregulation of the ADAMTS-4 proteoglycanase activity, particularly at the end stage of the disease in SOD1-G93A mice lumbar spinal cord. However, differently from spinal cord injury, the decrease of the metalloproteinase could be a protective tissue response to maintain a robust ECM envelope, with the aim to render neurons less vulnerable to degeneration (30), although it cannot be excluded that, the downregulation of ADAMTS-4 and the consequent thickening of the PNN could be a neuron regeneration-opposing process. With a supposed protective extent, semaphorin Sema3A, an extracellular matrix molecule mainly expressed by meningeal fibroblasts and involved in the inhibition of axonal degeneration, decreases progressively in SOD1-G93A mice spinal cord, highlighting the remodeling of the ECM as an attempt to rescue the inhibition of axonal regeneration and growth cone collapse (31). Elevated levels of connective tissue growth factor (CTGF) a protein involved in different processes, among which adhesion, migration, and synthesis of ECM components is increased in the spinal cord of ALS patients (32). Although it can be speculated that this protein may exert non-fibrotic roles in the CNS, i.e., interfering with oligodendrocyte maturation and proper axon myelination, it cannot be excluded that it could be involved in ECM remodeling function in the CNS of ALS patients.

**Figure 1:**
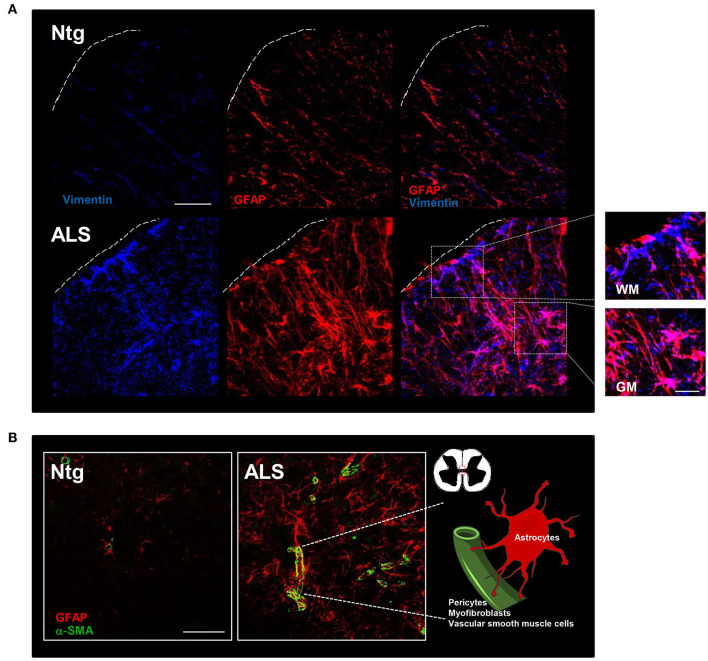
Fibrotic and glial scar in ALS mice. **(A)** Representative confocal images of lumbar spinal cord sections from non-transgenic (Ntg) and hFUS (ALS) mice at end stage of the disease. ALS section displays abundant vimentin-immunoreactive cells (blue) and glial fibrillary acidic protein (GFAP)-immunoreactive astroglial cells organized in a scar-like fashion (red). Scale bar = 20 μm. A higher magnification of the area marked by the white square is shown on the right and displays cells stained by both vimentin and GFAP in the gray matter (GM) and vimentin-positive cells closely associated to GFAP-positive astrocytes in the white matter (WM) Scale bar = 100 μm. **(B)** Representative confocal images of lumbar spinal cord sections from non-transgenic (Ntg) and hFUS (ALS) mice at end stage of the disease. ALS section displays an increase in α-smooth muscle actin (α-SMA)-immunoreactive cells (green) closely surrounded by GFAP-positive cells (red). Scale bar = 100 μm.

Although the remodeling of the ECM in ALS is attributed mainly to astrocytes (33, 34), other cell types can contribute to this phenomenon. Mesenchymal cells of meningeal and perivascular origin could have a major role in creating a fibrotic environment in the CNS. In this regard, S100A4, a member of the S100 Ca^2+^-binding protein family, is strongly upregulated in ALS models, starting from presymptomatic stages (35, 36) and its overexpression, mainly ascribable to spinal astrocytes and microglia (35), occurs likewise in other cell types. S100A4 is well-known to exhibit a pivotal role in promoting changes in cellular phenotype, as it is highly expressed in cells that are undergoing a mesenchymal transition or are converting into a more reactive state (37). Moreover, S100A4 favors ECM deposition in tissues, contributing to the scar formation (38). Therefore, the increase in S100A4 during ALS could be related to inflammation, fibrosis and tissue remodeling in disease progression. Furthermore, vimentin, a type III intermediate filament protein, shared by reactive astrocytes and mesenchymal cells, increases its expression in SOD1-G93A mice (39, 40), as well as in the spinal cord of symptomatic ALS transgenic mice overexpressing wild-type human FUS (hFUS) (Figure 1A). Together with S100A4, vimentin and fibronectin, α-smooth muscle actin (α-SMA) is commonly used to identify activated mesenchymal cells surrounding CNS blood vessels which are a major source of injury-induced myofibroblasts after damage (41, 42). In the spinal cord of ALS models, α-SMA increases in thickened blood vessels of SOD1-G93A mice (43) and in vessels in close proximity to astrocytes in the formation of the neurovascular unit of hFUS mice (Figure 1B). This evidence suggests that in ALS CNS there is an increase of mesenchymal cells, such as myofibroblasts derived from perivascular and endothelial cells, which could contribute to a pro-fibrotic environment. Indeed, elevated levels of various inflammatory cytokines in ALS patients could induce a pro-inflammatory endothelial cell response promoting the synthesis of monocyte-attracting chemokines and vascular cell-adhesion molecules, leading to parenchymal invasion of inflammatory and immune cells (44). Furthermore, the loss of pericytes in the blood-brain and blood-spinal cord barrier, a well-established pathogenic mechanism in ALS, correlated with the worsening of the disease, leads to accumulation of blood cells and proteins (such as immunoglobulin G, fibrin and thrombin) in the CNS (45). In a similar way to what occurs in AD (18), we can speculate that pericytes can actively contribute to the fibrotic scar by transforming into myofibroblasts.

## Fibro-Glial Scar in MS

Multiple sclerosis (MS) is a neuroinflammatory CNS disease where white matter axonal demyelination, accompanied by the disruption of the blood-brain barrier and consequent infiltration of monocyte-derived macrophages and lymphocytes, causes multiple white matter scars in the brain and spinal cord (46). A large amount of literature describes in both MS patients and experimental autoimmune encephalomyelitis (EAE) animal models, the formation of a fibrotic scar near the well-known glial scar. Particularly, in the active MS brain lesions and in EAE mouse spinal cord, besides the neuroinflammatory component, a widespread deposition of ECM, pro-fibrotic factors, collagens and fibronectin aggregates has been observed, all concurring to impair the remyelination process (8, 47). Moreover, biglycan and decorin are up-regulated in the demyelinated regions closely associated with immune cells infiltrates in the parenchyma (48). In addition, recent papers have shown the presence of brain mesenchymal perivascular aggregates of platelet-derived growth factor receptor (PDGFR)β-positive cells in MS patients and in the EAE model, where they contribute to fibrotic scar tissue generation, active inflammation and demyelination (49). In brain demyelinated plaques of MS patients and in leucocyte-containing perivascular cuff, an important portal where immune cells infiltrate into the CNS parenchyma, CSPGs are up-regulated. In this context they increase the activation and transmigratory capacity of macrophages and impair remyelination by interfering with the migration of pro-regenerative neural and oligodendrocyte precursor cells into lesions (50, 51).

The emerging novel function of microvascular endothelial cells in boosting secondary injury by promoting inflammation, microvessel dilation, and fibrotic scar formation is recently described by Zhou and co-authors in the EAE model. They show that endothelial cells exert critical functions beyond myelin clearance in promoting the progression of demyelination disorders, by regulating macrophage infiltration, pathologic angiogenesis and fibrosis. Particularly, they demonstrate that the engulfment of myelin debris induces a mesenchymal transition, transforming endothelial cells into a source of fibrotic molecules as collagen and fibronectin (52). Therefore, both inflammation and ECM deposition contribute to impair the regenerative ability of oligodendrocyte progenitor cells to replace mature oligodendrocytes, thus prolonging the demyelination of lesions. Recently, Yan and colleagues, by using Col1α1GFP transgenic mice to visualize scar-forming cells in the lesioned tissue, showed that perivascular fibroblasts are activated in the EAE model at the onset of the disease and infiltrate the parenchyma next to the areas of demyelination and the ECM deposition. Moreover, they showed that both fibroblast conditioned medium and fibroblast ECM hinder the differentiation of oligodendrocyte progenitor cells into mature oligodendrocytes (53).

## Fibro-Glial Scar in AD

Alzheimer's disease (AD) is a neurodegenerative disorder characterized by the formation of “plaques” constituted by an excess of fibrous tissue in the brain. Brain tissue from AD patients indeed shows extensive deposition of extracellular β-amyloid aggregates accumulating into toxic fibrils, along with several other extracellular molecules, including GAGs and proteoglycans, with a prevalence of heparan sulfate proteoglycans (HSPGs) (54). HSPGs exert a critical role in amyloid precursor protein cleavage and the resulting β-amyloid fibrillization (55, 56). Remarkably, the study of Garcia et al. (57) reports that the expression of heparanase, an endoglucuronidase that specifically degrades heparan sulfate (HS) side chains, is upregulated in the brain of AD patients both within intracellular deposits of degenerating neurons and in extracellular plaques. Yet, in the brains of mice overexpressing heparanase, the recruitment and activation of inflammatory cells are significantly attenuated as well as immune cell-mediated clearance of β-amyloid deposits, proving that intact HS chains are required to mediate neuroinflammatory responses and highlighting a possible beneficial role of HS in the disease (58, 59). These apparently contradictory results are nevertheless in line with the dual role of the fibrotic process particularly linked to the inflammatory responses activated during the progression of neurodegenerative pathologies. The importance of interfering with HSPGs is supported by novel studies showing that this mechanism could represent a possible therapeutic approach in the control of fibrotic and glial scarring in AD pathology. Consistently, an antibody targeting extracellular tau is able to potently inhibit its neuronal internalization by masking epitopes that are important for the interaction with neuron surface HSPGs, constituting therefore a potential strategy in hampering AD (60). Moreover, HSPGs-mediated tau internalization is inhibited by the sulfated glycosaminoglycan heparin that, however, is characterized by a low brain penetration and strong anticoagulant properties (61). Stopschinski et al. (62) recently developed a synthetic heparinoid devoid of anticoagulant activity able to inhibit tau binding to GAGs and consequently its cellular uptake.

An increasing attention is now given to the role of pericyte cells as an attractive target involved in the pathogenesis, progression and potential treatment of AD (18). In AD patients, pericyte density is associated with blood-brain barrier breakdown, reported to be strongly responsible for the cognitive deficits characterizing the disease and identified as an early biomarker. Moreover, pericytes and vascular smooth muscle cells constitute the major cellular phenotypes expressing PDGFRβ, the levels of which in the cerebrospinal fluid positively correlate with clinical dementia rating in AD patients (63).

## Conclusions

A fibrotic environment within the CNS can be a consequence of infections, parasite infestations, and neuronal injury. While acute brain and spinal cord traumas generate a well-defined fibrotic scar, where ECM, myofibroblasts, and astrocytes are clearly organized into a discernible structure, in neurodegenerative diseases the formation of a fibrotic environment in neuronal tissue is less obvious. However, a number of evidence indicates that in chronic situations of neuron loss, as those occurring in neurodegenerative conditions, there is a progressive replacement of damaged tissue with ECM components, mainly produced by activated fibroblasts and astrocytes and this leads to a secondary response involving microglia and peripheral immune cells. Nevertheless, the outcome of acute injury and the progression of chronic disease such as ALS, MS, and AD share most responder cells and many of the principal mediators and extracellular components, some of which have been described in this review and summarized in Table 1.

**Table 1:** Synoptic view of main features of acute and chronic CNS fibro-glial scar.

**CNS disease**	**Responder cells**	**Mediators**	**ECM component**
Acute damage	Astrocytes, microglia, leukocytes, meningeal cells, fibroblasts, pericytes	Thrombin, MMP-9, ATP, PDGFRβ, TGFβ	Fibronectin, laminin, collagen, CSPGs, tenascin, HSPGs
ALS	Astrocytes, microglia, leukocytes, oligodendrocytes, meningeal cells, fibroblasts, pericytes	IL-6, CXCL1, CXCL10, CXCL12, TNFα, TGFβ, NGF, INFγ, PGD2, ADAMTS-4, CTGF, S100A4, MMP-9	Fibronectin, collagen IV, CSPGs, Sema3A, fibrin, vimentin, thrombin
MS	Astrocytes, microglia, leukocytes, endothelial cells, meningeal cells, fibroblasts, pericytes, oligodendrocytes	PDGFRβ, TGFβ, myelin	Collagen, fibronectin, biglycan, decorin, CSPGs
AD	Astrocytes, microglia, leukocytes, smooth muscle cells, fibroblasts, pericytes	PDGFRβ, TGFβ	GAGs, HSPGs

*The table summarizes the main cellular components, mediators and ECM molecules involved in ALS, MS, and AD as well as in acute CNS damage. For a more extensive review of the many specific molecules that regulate or influence CNS cellular responses to acute conditions see (3, 4). CTGF, connective tissue growth factor; INFγ, interferon γ; MMP-9, matrix metalloprotease-9; NGF, nerve growth factor; PGD2, prostaglandin D2; TNFα, tumor necrosis factor α*.

Although the purpose of fibrotic material deposition is to limit damage spreading, the prolonged, and massive response that often characterizes this process eventually impedes tissue regeneration and axons preservation. Thus, while wound healing is an advisable event in neurodegenerative conditions, it would be therapeutically helpful to try to modify and resolve scarring toward a beneficial pro-axon regeneration feature, by manipulating for instance myofibroblasts, originating from perivascular or meningeal tissues or from pericytes, and glial cells, with the overall aim to improve the outcome of diseases (2). Since neurodegenerative diseases are mediated by multifactorial pathways and are characterized by multicellular responses, it is now clear that their successful treatment should necessarily be multi-targeted. Toward this end, strategies aimed at removing excessive fibrotic matrix and slowing-down the relentless deposition of ECM by reactive glia and fibroblasts, could be promising because they could limit the chronic inflammation that is associated to fibrosis.

By targeting the molecular mechanisms which are involved in the process of fibrotic scar formation as inflammatory responses, autophagy, debris uptake, and mesenchymal reactivity, it could be possible to reverse the effects of CNS associated fibrosis. In this regard, future directions could be represented by anti-fibrotic agents acting as novel potential therapeutic targets for the treatment of MS, re-establishing an environment where the remyelination is not hindered by the formation of a fibrotic scar, followed by the appearance of neuroinflammatory lesions. In ALS and AD, where both inflammation and fibrosis strongly contribute to the pathogenesis of the diseases, switching cells such as astrocytes and myofibroblasts from a matrix-depositing state that supports fibrosis to a matrix-degrading state that promotes resolution or reversal of fibrosis may contribute to ameliorate pathological conditions.

## Author Contributions

All authors listed have made a substantial, direct and intellectual contribution to the work, and approved it for publication.

## Conflict of Interest

The authors declare that the research was conducted in the absence of any commercial or financial relationships that could be construed as a potential conflict of interest.

## Abbreviations

AD: Alzheimer's disease
ALS: amyotrophic lateral sclerosis
CSPGs: chondroitin sulfate proteoglycans
EAE: experimental autoimmune encephalomyelitis
ECM: extracellular matrix
GFAP: glial fibrillary acidic protein
hFUS: human FUS
HS: heparan sulfate
HSPGs: heparan sulfate proteoglycans
MS: multiple sclerosis
PNN: perineuronal net
PDGFR: platelet-derived growth factor receptor β
α-SMA: α-smooth muscle actin
TGFβ: transforming growth factor (TGF)β.
